# The predictive value of peripheral blood cells and lymphocyte subsets in oesophageal squamous cell cancer patients with neoadjuvant chemoradiotherapy

**DOI:** 10.3389/fimmu.2022.1041126

**Published:** 2022-11-14

**Authors:** Jin Zhou, Hai-Ping Lin, Xin Xu, Xiao-Hang Wang, Ling Rong, Yao Zhang, Lei Shen, Lei Xu, Wei-Ting Qin, Qing Ye, Xiu-Mei Ma, Yong-Rui Bai

**Affiliations:** ^1^ Department of Radiation Oncology, Ren Ji Hospital, School of Medicine, Shanghai Jiao Tong University, Shanghai, China; ^2^ Department of Thoracic Surgery, Ren Ji Hospital, School of Medicine, Shanghai Jiao Tong University, Shanghai, China; ^3^ Department of Gastroenterology, Ren Ji Hospital, School of Medicine, Shanghai Jiao Tong University, Shanghai, China

**Keywords:** neoadjuvant chemoradiotherapy, pathological complete response, lymphocyte subsets, peripheral blood cell, oesophageal squamous cell cancer

## Abstract

**Purpose:**

Neoadjuvant chemoradiotherapy (nCRT) is a standard treatment option for patients with stage III oesophageal cancer. Approximately 30% of oesophageal cancer patients will have a pathological complete response (pCR) after nCRT. However, available clinical methods cannot accurately predict pCR for patients. We aimed to find more indicators that could be used to predict the pathological response to nCRT.

**Method:**

A total of 84 patients with stage III oesophageal squamous cell cancer were enrolled in this study. Ten patients failed to have surgery as a result of progressive disease (PD). Among the patients who underwent surgery, 32 patients had a pathologic complete response (pCR), whereas 42 patients showed no or partial response (npCR) after nCRT. Routine blood test results and lymphocyte subset assessments before and after nCRT were retrospectively analysed. Univariate and multivariate analyses were used to identify independent predictors of the clinical curative effect of nCRT. Eventually, nomograms were established for predicting the PD and pCR rates.

**Results:**

The numbers of lymphocytes, B lymphocytes, T lymphocytes, Th lymphocytes, Ts lymphocytes, and NK cells and the percentages of B lymphocytes and NK cells were decreased significantly after nCRT (*P* < 0.0001), whereas the percentages of T lymphocytes and Ts lymphocytes increased (*P* < 0.0001). Univariate analysis showed that age, the length of the lesion, the level of haemoglobin before nCRT, and the amount of change in haemoglobin were related to PD, and the percentage of NK cells after nCRT was related to pCR. Multivariate logistic analysis demonstrated that the length of the lesion, the neutrophil-to-lymphocyte ratio (NLR) before nCRT, and the amount of change in haemoglobin were independent predictors of PD, whereas the percentage of NK cells after nCRT was an independent predictor of pCR.

**Conclusion:**

Lymphocyte subsets changed dramatically during nCRT, and these changes together with baseline and posttreatment lymphocyte subsets have predictive value in determining the response to nCRT for oesophageal cancer.

## Background

Oesophageal carcinoma is one of the most familiar malignant tumours, with 604,100 new cases of oesophageal cancer worldwide every year (1). There are two main types of oesophageal cancer: squamous cell carcinoma and adenocarcinoma. Although the incidence of oesophageal adenocarcinoma now exceeds that of squamous cell carcinoma in the USA and some western European countries, squamous cell carcinoma is still the predominant histological type of oesophageal carcinoma in East Asia (2). Squamous cell carcinoma accounts for more than 90% of the total number of oesophageal carcinoma cases in China (3). Surgery is the main treatment for oesophageal cancer, but chemotherapy and radiotherapy also play an important role in oesophageal cancer treatment, especially for those whose tumours cannot be completely resected. Several clinical trials have demonstrated that neoadjuvant chemoradiotherapy can improve long-term survival and reduce locoregional cancer recurrence (4, 5). Approximately 30% of oesophageal cancer patients will have a pathological complete response (pCR) after neoadjuvant chemoradiotherapy, and these patients will benefit more from neoadjuvant chemoradiotherapy (6–8). In recent years, the question has been raised of whether surgery can be dispensed with for patients who achieve pCR. However, the main problem is that we cannot distinguish which patient has achieved pCR without surgery. If a method that was verified to exactly predict the efficacy of neoadjuvant chemoradiotherapy (nCRT) was available, it would be of great clinical significance to the formulation of treatment options for patients.

At present, the efficacy of nCRT is mainly evaluated by imaging. However, in some patients with complete response evaluated by imaging, residual cancer tissue was still found during surgery. Hence, researchers have been looking for new predictors for the efficacy of nCRT. The NLR is an indicator of inflammation that has been linked with the prognosis of oesophageal cancer and other tumours in many studies (9–16). Sun Yat-sen University Cancer Center retrospectively analysed 306 ESCC patients who underwent nCRT and found that the NLR before nCRT and the PLR after nCRT were independent predictors of pCR (17). Recently, a real-world study established a prognostic model based on the NLR, the prognostic nutrition index (PNI), eosinophilic granulocytes (EOS), and the postoperative pathologic stage of adenocarcinoma for oesophagogastric junction patients treated with neoadjuvant chemoradiotherapy (18). However, due to their accuracy, these models have not been used clinically. Therefore, it is necessary to find new predictors.

In this study, we aimed to investigate potential indicators associated with the efficacy of nCRT. In addition, we developed prognostic nomograms for the clinical curative effect of nCRT based on certain clinical features and immunity indices.

## Methods

### Patients

This retrospective study included 84 oesophageal cancer patients who received nCRT at Shanghai Renji Hospital between September 2014 and June 2021. The study inclusion criteria were as follows: 1) patients were first diagnosed with clinical stage III oesophageal cancer evaluated by enhanced CT, endoscopic ultrasonography, or PET-CT (American Joint Committee on Oncology, Edition 8); 2) receipt of neoadjuvant chemoradiotherapy; 3) availability of routine blood and lymphocyte subset examination within 1 week prior to and after treatment; 4) Karnofsky performance status ≥80. The study was approved by the Medical Ethics Committee of our institute, and all the patients provided written informed consent.

### Treatment

Intensity-modulated radiotherapy was used in all radiotherapy treatments, with a total dose of 41.4 Gy/23 Fx or 40 Gy/20 Fx. The gross tumour volume (GTV) was defined as the primary tumour and that of suspected metastatic lymph nodes visible on CT or PET-CT scans. Elective nodal irradiation was used in our study. The clinical target volume (CTV) consisted of a 3-cm expansion of the GTV at the proximal and distal margins and 5 mm in the radial direction, and the regional lymph nodes that were prophylactically irradiated were also included. The planning target volume (PTV) was generated from the CTV by adding a uniform margin of 5 mm.

Most patients in the study received concurrent intravenous chemotherapy during the radiotherapy, weekly or triweekly. Triweekly therapy refers to cisplatin 75 mg/m^2^ with paclitaxel 135 mg/m^2^ every 3 weeks for two cycles. Weekly therapy refers to cisplatin 25 mg/m^2^ with paclitaxel 45 mg/m^2^ every week for five cycles. Two patients received oral tegafur for concurrent chemotherapy. Then, patients received surgery about 6 weeks after completion of neoadjuvant chemoradiotherapy if they had no disease progression in the preoperative radiographic evaluation.

### Response evaluation

Patients were divided into the PD group, pCR group (no residual tumour at the primary site or lymph nodes, ypT0N0M0), and no or partial response (npCR) group (residue was found in the primary lesion or lymph nodes) based on radiographic and pathological evaluation. Ten patients did not receive surgical treatment at the end of nCRT as a result of PD.

### Data collection

The location of each tumour was determined by the centre of the tumour according to the American Joint Committee on Oncology, Edition 8. Lesion length refers to the length measured directly under endoscopy. The results of routine blood and lymphocyte subset tests before and after neoadjuvant chemoradiotherapy were collected. The following research indicators were included in this study: NLR, platelet-to-lymphocyte ratio (PLR), and immune-inflammation index (SII). The NLR, PLR, and SII were calculated as follows:

NLR = lymphocyte count ÷ neutrophil count
PLR = platelet count ÷ lymphocyte count
SII = platelet count × neutrophil count ÷ lymphocyte count.

### Statistical analysis

Continuous variables, such as age and lesion length, were converted into binary variables using cut-off values calculated from receiver operating characteristic (ROC) curves. Univariate logistic regression analysis was used to estimate the odds ratio (OR) and confidence interval (CI) to evaluate the effect of independent variables on the clinical curative effect of nCRT. To avoid omitting indicators that might be of clinical significance, all possible factors were subjected to multivariate analysis, and the backwards method was used to establish the best multivariate analysis regression model. The multivariate logistic regression model is presented in the form of a line graph. Then, the prediction model was evaluated by internal random sampling. A significant difference was considered if the two-sided *P* value was < 0.05. Statistical analysis was performed with IBM SPSS Statistics 25.0. A nomogram for possible prognostic factors was established with R 4.0.3, and the predictive accuracy was evaluated using the concordance index (c-index).

## Results

### Patient characteristics

A total of 84 patients with oesophageal cancer were enrolled in the study, with an overall pCR rate of 38.1% (32/84). All patients were first diagnosed with stage III oesophageal squamous cell carcinoma, including 71 males (84.5%) and 13 females (15.5%). The median age of the patients was 64 years, ranging from 48 to 79 years. Almost all patients (except one patient receiving a dose of 40 Gy/20 Fx) were scheduled to receive a standard radiation dose of 41.4 Gy/23 Fx, but two patients failed to complete the radiotherapy plan. One patient only completed 39.6 Gy/22 Fx, and another only completed 37.8 Gy/21 Fx; both failed to achieve a pCR.

All patients received concurrent chemotherapy. Triweekly therapy was selected by 33 patients, 39.4% (13 patients) of whom achieved a pCR, whereas among the other 49 patients who received weekly therapy, 17 (34.7%) patients achieved a pCR. The remaining two patients received oral tegafur, and both of them achieved pCR. All patients received surgical treatment about 6 weeks after the end of nCRT, except for the 10 patients (11.9%) who experienced PD and had tumours that were unable to be completely removed. According to the surgical pathology, patients were divided into a pathological complete response group and partial or no response group. Additional information about patient demographics, tumour characteristics, and treatment regimens are listed in **Tables 1**, **2**.

**Table 1:** Patient characteristics between inoperable and operative groups.

Characteristic	Operation	Inoperable	*P* value	Univariate	
	(n = 74)	(n = 10)		Odds ratio (95% CI)	*P* value
Age			0.001		
≤70	61	3		Reference	
>70	13	7		10.95 (2.495-48.054)	0.002
Gender			0.292		
Male	61	10		Reference	
Female	13	0		1.000	0.993
T stage			1.000		
T1-2	4	0		Reference	
T3-4	69	10		1.000	0.994
N stage			1.000		
N0	3	0		Reference	
N1-2	70	10		1.000	0.995
Location			0.540		
Upper	9	2		Reference	
Middle	27	2		0.333 (0.041-2.722)	0.305
Lower	38	6		0.711 (0.123-4.120)	0.703
Length of the lesion			0.027		
≤6.75 cm	58	4		Reference	
>6.75 cm	16	6		5.438 (1.391-23.570)	0.016
Chemotherapy			0.323		
Triweekly	31	2		Reference	
Weekly	41	8		1.000	0.180
Tegafur	2	0		1.000	0.999

**Table 2:** Patient characteristics between pCR and npCR groups.

Characteristic	pCR	nPCR	*P* value	Univariate	
	(n = 32)	(n = 42)		Odds ratio (95% CI)	*P* value
Age			0.489		
≤70	28	33		Reference	
>70	4	9		0.52 (0.146-1.886)	0.322
Gender			1.000		
Male	26	34		Reference	
Female	6	7		1.15 (0.347-3.841)	0.816
T stage			0.424		
T1-2	3	1		Reference	
T3-4	29	40		0.24 (0.024-2.442)	0.229
N stage			0.809		
N0	2	1		Reference	
N1-2	30	40		0.38 (0.032-4.331)	0.432
Location			0.182		
Upper	4	5		Reference	
Middle	8	19		0.53 (0.111-2.487)	0.418
Lower	20	18		1.39 (0.322-5.986)	0.659
Length of the lesion			1.000		
≤6.75 cm	25	33		Reference	
>6.75 cm	7	9		1.03 (0.336-3.134)	0.963
Chemotherapy			0.332		
Triweekly	31	2		Reference	
Weekly	41	8		1.000	0.180
Tegafur	2	0		1.000	0.993

### Changes in peripheral blood lymphocyte subsets in oesophageal cancer patients after nCRT

**Figure 1:**
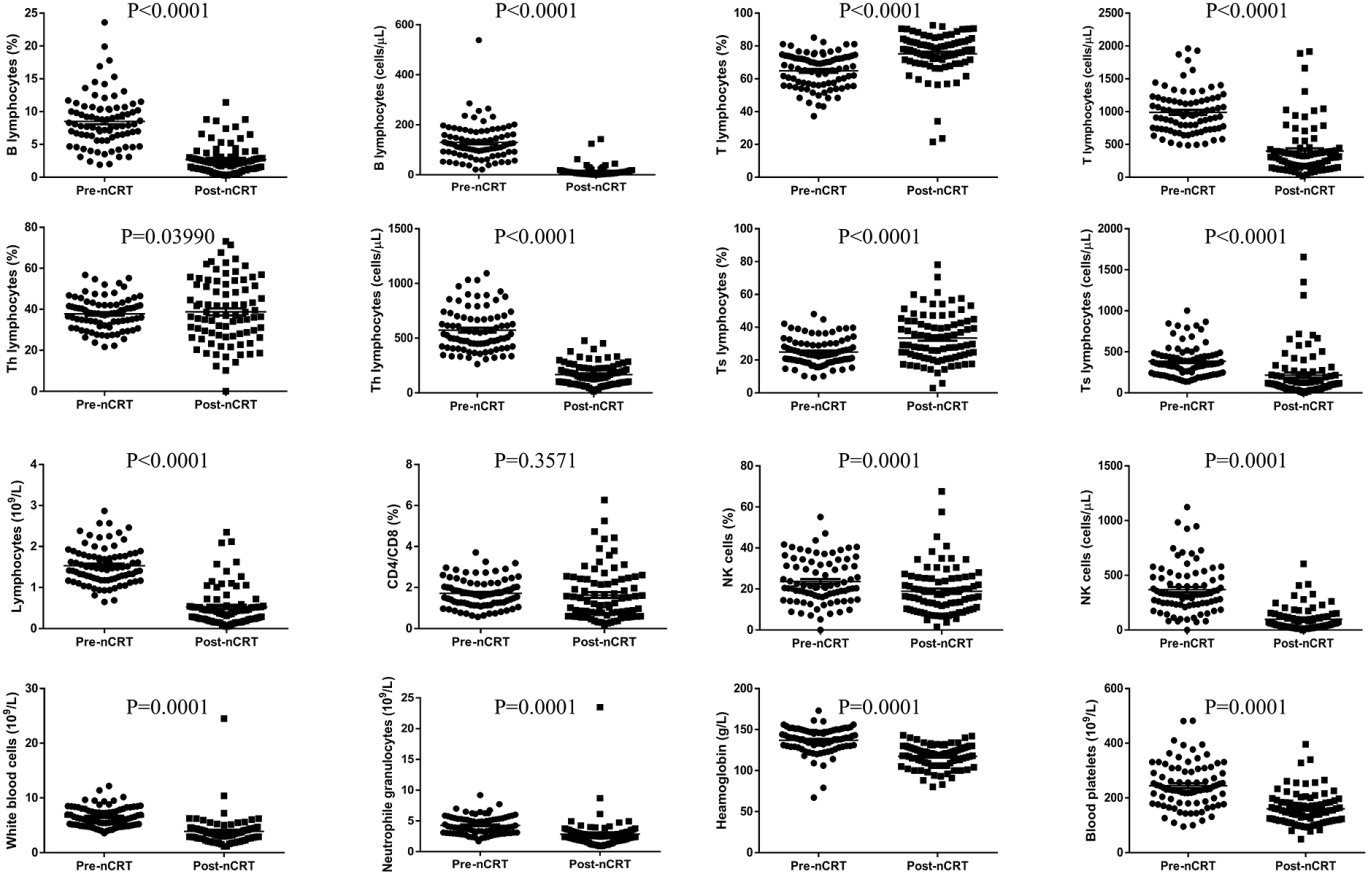
Changes in lymphocyte subsets, peripheral blood cells, and haemoglobin.

The proportion of peripheral blood lymphocyte subsets is shown in **Figure 1** and **Table 3**. The results indicated that the numbers of lymphocytes, B lymphocytes, T lymphocytes, Th lymphocytes, Ts lymphocytes, and NK cells and the percentages of B lymphocytes and NK cells were decreased significantly after nCRT (*P* < 0.05), whereas the percentages of T lymphocytes and Ts lymphocytes increased (*P* < 0.05). Notably, only the CD4/CD8 value and the percentage of Th lymphocytes did not show significant differences before and after nCRT (*P* > 0.05). These results suggest that nCRT treatment led to changes in the composition of peripheral blood lymphocyte subsets.

**Table 3:** Changes in the lymphocyte subset before and after nCRT.

Lymphocyte subset	Value (median)		*P* value
	Before nCRT	After nCRT	
Lymphocytes (10^9^/L)	1.47	0.435	<0.0001
B lymphocytes (%)	8.00	2.05	<0.0001
B lymphocytes (cells/µL)	118.45	8.60	<0.0001
T lymphocytes (%)	66.30	77.40	<0.0001
T lymphocytes (cells/µL)	944.25	304.75	<0.0001
Th lymphocytes (%)	37.90	37.05	0.3990
Th lymphocytes (cells/µL)	532.95	143.20	<0.0001
Ts lymphocytes (%)	23.05	29.45	<0.0001
Ts lymphocytes (cells/µL)	346.70	110.40	<0.0001
CD4/CD8	1.665	1.230	0.3571
NK cells (%)	20.75	16.85	0.0001
NK cells (cells/µL)	324.65	62.45	0.0001
WBCs (10^9^/L)	6.195	3.525	0.0001
Neutrophile granulocytes (10^9^/L)	3.975	2.505	0.0001
Haemoglobin (10^9^/L)	138.00	119.50	0.0001
Blood platelets (10^9^/L)	231.00	147.50	0.0001

### Factors associated with efficacy of nCRT

**Figure 2:**
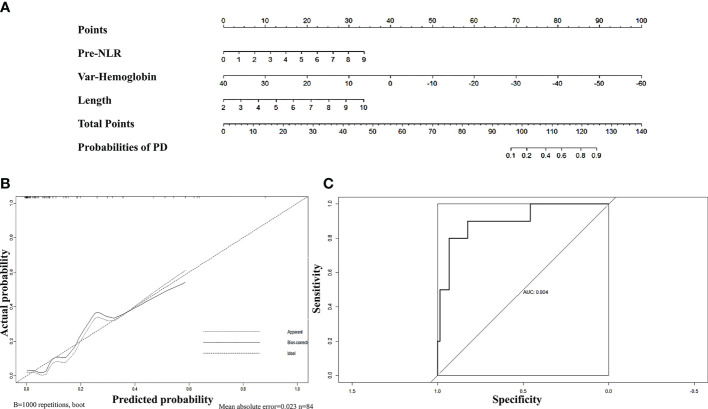
**(A)** Nomogram for predicting PD rates after neoadjuvant chemoradiotherapy in patients with oesophageal squamous cell carcinoma. The nomogram adds up the points identified on the scale for each independent factor. The total scores projected on the bottom scale indicate the probabilities of PD. **(B)** Calibration plots of the nomograms. **(C)** ROC curves of the nomogram.

**Figure 3:**
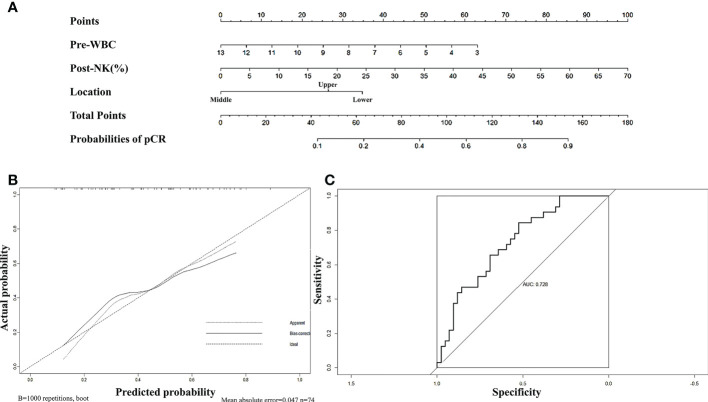
**(A)** Nomogram for predicting pCR rates after neoadjuvant chemoradiotherapy in patients with oesophageal squamous cell carcinoma. The nomogram adds up the points identified on the scale for each independent factor. The total scores projected on the bottom scale indicate the probabilities of pCR. **(B)** Calibration plots of the nomograms. **(C)** ROC curves of the nomogram.

First, we sought to explore whether lymphocyte subsets combined with clinical characteristics could be used to predict PD. The results of univariate analysis suggested that age, the length of the lesion, the level of haemoglobin before nCRT, and the amount of change in haemoglobin may be related to PD (**Supplementary table 1**). Factors with a univariate significance of *P* < 0.1 were enrolled in multivariate analysis. Multivariate logistic analysis further demonstrated that the length of the lesion, the NLR before nCRT, and the amount of change in haemoglobin were independent predictors of PD (**Table 4**). A nomogram was developed to predict the risk of PD on the basis of multivariate logistic regression coefficients (**Figure 2A**). The C-index of the nomogram was 0.90 (95% CI, 0.797 to 1.000), and the calibration curve displayed acceptable agreement between prediction and actual observation (**Figures 2B, C**). Then, we further explored the factors associated with pCR among the patients who received surgery after nCRT. In univariate analysis (**Supplementary table 2**), we found that only the percentage of NK cells after nCRT was related to pCR (*P* = 0.04, OR, 1.047, 95% CI, 1.005–1.098). We relaxed the inclusion criteria, and factors with a univariate significance of *P* < 0.5 were enrolled in the multivariate analysis. The results of the multivariate analysis are shown in **Table 5**. Similarly, a nomogram to predict the risk of pCR was developed on the basis of multivariate logistic regression coefficients (**Figure 3A**). The C-index of the nomogram was 0.73 (95% CI, 0.614 to 0.843), and the calibration curve displayed acceptable agreement between the prediction and actual observation (**Figures 3B, C**).

**Table 4:** Multivariate analysis of PD.

Variable	Multivariate analysis	
	Odds ratio (95% CI)	*P* value
Length	0.526 (0.304-0.910)	0.022
Var-haemoglobin	1.166 (1.060-1.281)	0.001
Pre-NLR	0.564 (0.295-1.078)	0.083

**Table 5:** Multivariate analysis of pCR.

Variable	Multivariate analysis	
	Odds ratio (95% CI)	*P* value
Pre-WBC	0.778 (0.590-1.028)	0.077
Post-NK cells (%)	1.058 (1.012-1.107)	0.013
Location		
Lower	Reference	Reference
Upper	0.719 (0.142-3.632)	0.698
Middle	0.252 (0.078-0.810)	0.021

## Discussion

nCRT is a standard presurgical treatment option for patients with stage III oesophageal cancer. Most patients can benefit from nCRT, making them more likely to obtain R0 resection. The optimal outcome is a complete pathological response. These oesophageal cancer patients with a pCR have a significantly improved overall survival (OS) (6). Recently, the question has been raised of whether surgery can be dispensed with for patients who achieve pCR. However, there is no method to predict pCR without surgery. Therefore, finding new predictors of the efficacy of nCRT is very meaningful and is helpful in preserving organ function.

Inflammation is a well-recognized cancer risk factor that substantially contributes to the development and progression of malignancies (19, 20). Tumours are infiltrated by inflammatory cells, whose numbers become an independent factor affecting patient outcomes (21, 22). A great number of studies have focused on immune cell infiltration in the tumour microenvironment (23–25), whereas the peripheral blood lymphocyte subsets in tumour patients have not been well studied.

The maintenance of a normal immune state depends on the coordination of various immune cells, especially peripheral lymphocyte subsets. The number of lymphocyte subsets is relatively constant, and this balance is upset in a pathological state. Circulating NK and regulatory T-cell proportions have been found to be significantly lower in patients with advanced gastric cancer (26). A prospective study longitudinally analysed the peripheral blood samples of 26 patients with solid tumours and found that patients who did not respond to chemotherapy or had only a slight response had a significant decrease in total B-lymphocyte counts, suggesting that the number of B cells in peripheral blood may be used to predict the effect of chemotherapy (27). In a retrospective cohort study of non-small cell lung cancer, a high baseline absolute CD4+ T lymphocyte count contributed to longer progression-free survival (28). In oesophageal cancer, it was also found that a high proportion of CD4+CD8+ (>3.45%) cells and a low proportion of regulatory T cells (≤5.15%) before chemoradiotherapy were related to a better OS. To date, no studies have revealed the relationship between circulating lymphocyte subsets and the efficacy of nCRT for oesophageal cancer. Thus, our study might provide novel indicators to predict the efficacy of nCRT.

In this study, 84 oesophageal cancer patients who underwent nCRT were enrolled. The results showed that the numbers of lymphocytes, B lymphocytes, T lymphocytes, Th lymphocytes, Ts lymphocytes, and NK cells and the percentages of B lymphocytes and NK cells were decreased significantly after nCRT, suggesting that these cells may play an important role in the treatment. We first compared the 10 patients whose disease progressed with the 74 other patients who underwent surgery, and the multivariate logistic analysis demonstrated that the length of the lesion, the NLR before nCRT, and the amount of change in haemoglobin were independent predictors of PD. This is consistent with most previous studies, indicating that a high NLR is indeed an adverse factor for nCRT (12, 13, 16, 29). Then, we turned our attention to the pCR group. pCR is harder to predict, as most variables, including the NLR and PLR, did not differ significantly between patients with a complete response and those with a partial response. Ultimately, univariate logistic analysis showed that only the percentage of NK cells after nCRT was positively correlated with pCR. Multivariate logistic analysis further demonstrated that the percentage of NK cells after nCRT was an independent predictor of pCR.

Of note, the model we established to predict PD had a high coincidence index of 0.90, and the model suggested that a higher NLR and longer lesion length before treatment and high levels of decreased haemoglobin during treatment increased the risk of PD. Consistent with most previous studies (12, 13, 16, 29), a high NLR is indeed a risk factor for PD. In addition, decreased haemoglobin seemed to be another important risk factor, as only one of the 50 patients (2%) with a haemoglobin decrease less than 23 g/l (cut-off value) was evaluated as having PD, whereas this proportion increased to 26.5% in the 34 patients with a haemoglobin decrease over 23 g/l. Lesion length is also a well-known risk factor. In our study, among those with a lesion length less than 6.75 cm (cut-off value), only 6% of patients were evaluated as having PD, whereas this number increased to 27% among patients with a lesion length over 6.75 cm. Perhaps further study can focus on whether patients with a high risk of PD can benefit from an increase in the intensity of nCRT.

There are also some limitations in this study. First, in our predictive model of pCR, upper and lower oesophageal carcinomas seem to be more prone to pCR than middle oesophageal carcinomas. One possible reason is that the number of patients with upper and middle oesophageal cancer was relatively small, and selection bias existed. Whether there is a relationship between lesion location and efficacy should be explored by further increasing the sample size. Second, the NLR was not an accurate predictor of pCR in our study, although it was indeed lower in the pCR group. A previous study also showed that the NLR had no predictive value for the pathologic response to nCRT (30). One common deficiency of this previous study and our study is that the number of samples was not large. Perhaps a difference in the NLR between the pCR group and the npCR group would be observable with an increase in sample size. Third, almost all patients had leukopenia of varying degrees during the treatment and used drugs to raise their white blood cell count, which inevitably influenced the peripheral blood lymphocyte results after nCRT. Testing multiple times during treatment to select the lowest level may be a method to reduce this interference. Fourth, despite being cost-effective and non-invasive, the discriminatory ability of the nomogram to predict pCR is still not sufficient to fully guide clinical decision-making. Thus, integrating other novel predictive factors, such as imaging data, nutritional status, and other biological indicators, into the model is needed to improve its prediction ability.

## Data availability statement

The raw data supporting the conclusions of this article will be made available by the authors, without undue reservation.

## Ethics statement

The studies involving human participants were reviewed and approved by Ethics Committee of Renji Hospital. The patients/participants provided their written informed consent to participate in this study.

## Author contributions

X-MM, Y-RB, and QY designed this study. JZ, H-PL, XX, and X-HW analysed the data. JZ, H-PL, and XX wrote the manuscript. LR, YZ, LS, LX, and W-TQ collected the data. X-MM and Y-RB revised the manuscript. All authors contributed to the article and approved the submitted version.

## Funding

This study was supported by the Science and Technology Commission of Shanghai Municipality (21ZR1438500), the Incubating Program for Clinical Innovation of Renji Hospital (PYDY-DZX-009), and the Beijing Xisike Clinical Oncology Research Foundation (Y-XD202001/zb-0011).

## Conflict of interest

The authors declare that the research was conducted in the absence of any commercial or financial relationships that could be construed as a potential conflict of interest.

## Publisher’s note

All claims expressed in this article are solely those of the authors and do not necessarily represent those of their affiliated organizations, or those of the publisher, the editors and the reviewers. Any product that may be evaluated in this article, or claim that may be made by its manufacturer, is not guaranteed or endorsed by the publisher.
