# Supplementary material for: The predictive value of peripheral blood cells and lymphocyte subsets in oesophageal squamous cell cancer patients with neoadjuvant chemoradiotherapy
Source: Front Immunol. 2022 Nov 14;13:1041126. doi: 10.3389/fimmu.2022.1041126 (PMC9701713; doi:10.3389/fimmu.2022.1041126)
Supplement: Supplementary file 1 [file Table_1.docx]

**Supplementary table 1**

| **Characteristic** | **Operation** | **Inoperable** | **Univariate** | |
| --- | --- | --- | --- | --- |
|  | **（n=74）** | **(n=10)** | **Odds ratio (95% CI)** | ***p*-value** |
| Pre-Lymphocytes (10^9^/L) | 1.49 (1.21 ,1.79) | 1.36 (1.17 ,1.55) | 0.521(0.092-2.314) | 0.421 |
| Pre-B lymphocytes (%) | 8.00 (6.15 ,10.40) | 7.90 (6.85 ,9.57) | 0.927(0.752-1.102) | 0.435 |
| Pre-B lymphocytes (cells/µL) | 121.35 (82.22 ,175.98) | 109.45 (97.77 ,132.00) | 0.994(0.981-1.004) | 0.315 |
| Pre-T lymphocytes (%) | 65.55 (56.10 ,73.05) | 72.35 (63.65 ,73.18) | 1.046(0.979-1.130) | 0.210 |
| Pre-T lymphocytes(cells/µL) | 944.25 (729.82 ,1181.45) | 942.25 (861.85 ,1123.50) | 1.000(0.998-1.002) | 0.860 |
| Pre-Th lymphocytes (%) | 37.90 (32.15 ,42.18) | 35.70 (31.67 ,38.18) | 0.956(0.869-1.004) | 0.311 |
| Pre-Th lymphocytes (cells/µL) | 543.95 (424.65 ,704.00) | 477.80 (392.50 ,590.68) | 0.997(0.992-1.001) | 0.184 |
| Pre-Ts lymphocytes (%) | 23.05 (19.08 ,29.45) | 25.15 (19.20 ,37.38) | 1.040(0.962-1.122) | 0.309 |
| Pre-Ts lymphocytes (cells/µL) | 346.70 (248.22 ,446.22) | 346.75 (215.95 ,524.05) | 1.000(0.996-1.003) | 0.880 |
| Pre-CD4/CD8 | 1.67 (1.26 ,2.18) | 1.50 (0.93 ,1.94) | 0.835(0.288-2.160) | 0.721 |
| Pre-NK cells (%) | 21.00 (17.03 ,32.95) | 18.05 (13.75 ,24.18) | 0.962(0.894-1.025) | 0.258 |
| Pre-NK cells (cells/µL) | 329.55 (247.10 ,482.82) | 242.85 (156.22 ,323.40) | 0.998(0.994-1.001) | 0.257 |
| Pre-WBC | 5.97 (4.92, 7.89) | 7.09 (5.98, 7.71) | 1.190(0.830-1.690) | 0.326 |
| Pre-Lymphocytes | 1.52 (1.28, 1.81) | 1.32 (1.18, 1.58) | 0.506(0.095-2.105) | 0.383 |
| Pre-Neutrophils | 3.77 (3.04, 5.30) | 5.02 (4.10, 5.30) | 1.325(0.850-2.070) | 0.204 |
| Pre-Hemoglobin | 137.00 (129.00, 146.00) | 150.50 (148.25, 156.00) | 1.155(1.066-1.294) | 0.003 |
| Pre-platelet | 231.00 (180.25, 303.50) | 227.50 (190.00, 280.75) | 0.999(0.909-1.007) | 0.815 |
| Pre-NLR | 2.57 (2.17, 3.18) | 3.35 (2.69, 4.41) | 1.462(0.910-2.373) | 0.103 |
| Pre-PLR | 59.74 (46.07, 72.09) | 49.88 (36.91, 63.29) | 0.973(0.934-1.005) | 0.138 |
| Pre-SII | 87.59 (67.84, 121.22) | 62.50 (54.35, 98.55) | 0.985(0.963-1.001) | 0.125 |
| Post-Lymphocytes (10^9^/L) | 0.44 (0.24 ,0.54) | 0.26 (0.18 ,0.50) | 0.839(0.132-3.084) | 0.819 |
| Post-B lymphocytes (%) | 2.20 (1.33 ,3.40) | 1.40 (0.67 ,2.18) | 0.744(0.420-1.087) | 0.218 |
| Post-B lymphocytes (cells/µL) | 9.05 (3.73 ,15.95) | 4.85 (3.45 ,6.58) | 0.913(0.790-0.999) | 0.137 |
| Post-T lymphocytes (%) | 76.35 (69.40 ,82.45) | 80.70 (76.28 ,84.18) | 1.068(0.994-1.172) | 0.121 |
| Post-T lymphocytes(cells/µL) | 312.40 (175.93 ,404.22) | 190.85 (150.73 ,407.63) | 1.000(0.998-1.002) | 0.981 |
| Post-Th lymphocytes (%) | 37.95 (28.20 ,52.15) | 33.80 (25.68 ,39.00) | 0.976(0.932-1.020) | 0.293 |
| Post-Th lymphocytes (cells/µL) | 168.90 (93.23 ,227.22) | 100.05 (67.53 ,144.65) | 0.995(0.986-1.002) | 0.231 |
| Post-Ts lymphocytes (%) | 29.10 (21.20 ,41.72) | 39.75 (29.88 ,50.18) | 1.042(0.997-1.091) | 0.068 |
| Post-Ts lymphocytes (cells/µL) | 112.70 (51.62 ,225.32) | 100.35 (51.05, 226.17) | 1.000(0.998-1.002) | 0.664 |
| Post-CD4/CD8 | 1.48 (0.68 ,2.46) | 0.81 (0.56 ,1.27) | 0.499(0.178-1.018) | 0.111 |
| Post-NK cells (%) | 17.70 (9.93 ,25.55) | 13.60 (10.53 ,18.95) | 0.952(0.871-1.018) | 0.217 |
| Post-NK cells (cells/µL) | 75.45 (29.92 ,123.95) | 40.50 (23.65 ,58.02) | 0.997(0.987-1.004) | 0.502 |
| Post-WBC | 3.50 (2.80, 4.39) | 3.52 (2.71, 4.36) | 1.027(0.747-1.239) | 0.809 |
| Post-Lymphocytes | 0.41 (0.22, 0.54) | 0.29 (0.19, 0.56) | 1.002(0.180-3.571) | 0.998 |
| Post-Neutrophils | 2.49 (1.85, 3.13) | 2.70 (1.88, 3.38) | 1.034(0.737-1.255) | 0.759 |
| Post-Hemoglobin | 119.50 (106.75, 128.75) | 119.00 (107.75, 125.25) | 0.998(0.952-1.049) | 0.925 |
| Post-platelet | 151.00 (120.00, 188.50) | 124.00 (113.25, 180.00) | 0.993(0.978-1.005) | 0.333 |
| Post-NLR | 6.42 (3.30, 11.51) | 9.91 (5.41, 10.95) | 1.001(0.923-1.052) | 0.979 |
| Post-PLR | 70.92 (46.06, 93.08) | 60.90 (43.86, 66.73) | 0.984(0.961-1.007) | 0.201 |
| Post-SII | 23.11 (12.20, 46.56) | 14.81 (10.54, 25.54) | 0.992(0.962-1.010) | 0.473 |
| Var-Lymphocytes (10^9^/L) | -1.07 (-1.36, -0.67) | -1.02 (-1.15, -0.99) | 1.264(0.411-3.578) | 0.667 |
| Var-B lymphocytes (%) | -5.15 (-8.38, -3.60) | -5.25 (-8.30, -3.48) | 1.005(0.851-1.188) | 0.953 |
| Var -B lymphocytes (cells/µL) | -113.70 (-165.22, -67.28) | -100.40 (-122.60, -93.12) | 1.004(0.994-1.015) | 0.501 |
| Var -T lymphocytes (%) | -11.00 (2.82, 18.50) | 8.05 (3.28, 18.35) | 1.012(0.964-1.072) | 0.658 |
| Var -T lymphocytes(cells/µL) | -637.85 (-817.95, -355.20) | -780.05 (-823.85, -622.18) | 1.000(0.999-1.001) | 0.883 |
| Var -Th lymphocytes (%) | 2.10 (-9.78, 13.82) | -0.35 (-7.33, 3.32) | 0.989(0.950-1.031) | 0.583 |
| Var -Th lymphocytes (cells/µL) | -375.60 (-553.45, -284.38) | -399.25 (-455.28, -319.62) | 1.001(0.998-1.005) | 0.435 |
| Var -Ts lymphocytes (%) | 4.65 (-0.88, 17.60) | 5.95 (3.57, 11.15) | 1.035(0.986-1.084) | 0.144 |
| Var -Ts lymphocytes (cells/µL) | -184.30 (-319.40, -93.65) | -242.20 (-357.43, -167.88) | 1.000(0.998-1.002) | 0.764 |
| Var -CD4/CD8 | 0.02 (-0.78, 0.52) | -0.23 (-0.44, -0.06) | 0.578(0.292-1.078) | 0.094 |
| Var -NK cells (%) | -5.95 (-11.50, 1.02) | -4.10 (-9.30, -0.12) | 0.995(0.935-1.055) | 0.866 |
| Var -NK cells (cells/µL) | -244.15 (-386.38, -131.65) | -173.80 (-246.62, -119.20) | 1.001(0.998-1.005) | 0.410 |
| Var-WBC | -2.56 (-3.96, -1.72) | -2.99 (-4.42, -2.63) | 0.933(0.664-1.198) | 0.656 |
| Var-Lymphocytes | -1.14 (-1.38, -0.72) | -1.06 (-1.16, -0.95) | 1.460(0.486-4.173) | 0.484 |
| Var-Neutrophils | -1.52 (-2.67, -0.63) | -1.51 (-3.25, -1.33) | 0.927(0.616-1.197) | 0.666 |
| Var-Hemoglobin | -17.00 (-27.00, -11.00) | -36.50 (-42.75, -26.50) | 0.890(0.820-0.950) | 0.002 |
| Var-platelet | -80.00 (-127.00, -40.50) | -86.50 (-143.50, -74.00) | 0.996(0.985-1.007) | 0.505 |
| Var-NLR | 3.98 (0.78, 9.19) | 6.94 (2.98, 7.90) | 0.994(0.910-1.048) | 0.867 |
| Var-PLR | 8.51 (-11.24, 26.78) | 9.75 (-10.27, 23.47) | 0.998(0.976-1.020) | 0.863 |
| Var-SII | -59.13 (-93.83, -36.56) | -53.42 (-74.17, -44.78) | 1.006(0.994-1.018) | 0.355 |

**Supplementary table 2**

| Characteristic | npCR | pCR | Univariate | |
| --- | --- | --- | --- | --- |
|  | (n=42) | （n=32） | Odds ratio (95% CI) | *p*-value |
| Pre-Lymphocytes (10^9^/L) | 1.50 (1.27-1.84) | 1.46 (1.19-1.76) | 0.775(0.276-2.095) | 0.617 |
| Pre-B lymphocytes (%) | 8.00 (6.32-10.55) | 7.90 (5.60-9.98) | 0.944(0.832-1.060) | 0.345 |
| Pre-B lymphocytes (cells/µL) | 118.45 (87.75-184.37) | 122.90 (79.77-143.98) | 0.995(0.998-1.002) | 0.182 |
| Pre-T lymphocytes (%) | 65.55 (58.05-73.08) | 63.90 (55.58-72.75) | 0.979(0.935-1.024) | 0.360 |
| Pre-T lymphocytes(cells/µL) | 950.50 (780.27-1211.52) | 936.15 (678.12-1150.55) | 0.999(0.997-1.001) | 0.432 |
| Pre-Th lymphocytes (%) | 38.80 (32.55-42.00) | 36.55 (29.35-44.22) | 0.992(0.934-1.054) | 0.802 |
| Pre-Th lymphocytes (cells/µL) | 560.85 (448.72-722.45) | 496.40 (412.50-692.40) | 0.999(0.997-1.001) | 0.487 |
| Pre-Ts lymphocytes (%) | 23.30 (19.60-29.98) | 22.00 (18.82-28.33) | 0.985(0.926-1.045) | 0.612 |
| Pre-Ts lymphocytes (cells/µL) | 346.40 (262.22-445.98) | 353.85 (219.38-474.45) | 1.000(0.997-1.002) | 0.710 |
| Pre-CD4/CD8 | 1.56 (1.26-2.04) | 1.72 (1.14-2.19) | 1.188(0.580-0.637) | 0.637 |
| Pre-NK cells (%) | 20.45 (14.97-30.95) | 24.50 (18.65-34.45) | 1.027(0.985-1.074) | 0.221 |
| Pre-NK cells (cells/µL) | 320.75 (247.30-418.08) | 347.70 (240.57-503.22) | 1.001(0.999-1.003) | 0.480 |
| Pre-WBC | 6.47 [5.14, 8.21] | 5.44 [4.70, 7.45] | 0.837(0.635-1.080) | 0.184 |
| Pre-Lymphocytes | 1.52 [1.27, 1.87] | 1.55 [1.28, 1.78] | 0.825(0.305-2.157) | 0.696 |
| Pre-Neutrophils | 4.00 [3.36, 5.56] | 3.27 [2.93, 4.99] | 0.808(0.572-1.111) | 0.203 |
| Pre-Hemoglobin | 138.50 [129.00, 146.75] | 134.00 [126.00, 143.50] | 0.991(0.960-1.022) | 0.562 |
| Pre-platelet | 241.00 [181.25, 321.25] | 227.50 [169.00, 292.00] | 0.996(0.990-1.002) | 0.215 |
| Pre-NLR | 2.54 [2.26, 3.16] | 2.57 [1.99, 3.20] | 0.802(0.507-1.186) | 0.299 |
| Pre-PLR | 61.67 [46.42, 68.40] | 57.10 [45.76, 75.37] | 1.005(0.985-1.025) | 0.627 |
| Pre-SII | 85.73 [67.84, 121.22] | 90.51 [68.99, 118.14] | 1.001(0.993-1.010) | 0.737 |
| Post-Lymphocytes (10^9^/L) | 0.45 (0.24-0.58) | 0.38 (0.24-0.53) | 0.845(0.281-2.346) | 0.749 |
| Post-B lymphocytes (%) | 2.20 (1.60-3.40) | 2.00 (1.28-3.20) | 0.888(0.698-1.095) | 0.288 |
| Post-B lymphocytes (cells/µL) | 10.00 (4.73-15.95) | 8.10 (2.98-15.77) | 0.988(0.957-1.010) | 0.360 |
| Post-T lymphocytes (%) | 77.55 (71.75-82.25) | 74.90 (67.38-83.00) | 0.986(0.950-1.021) | 0.425 |
| Post-T lymphocytes(cells/µL) | 339.45 (181.30-451.55) | 298.95 (171.32-367.45) | 1.000(0.998-1.001) | 0.612 |
| Post-Th lymphocytes (%) | 37.40 (27.02-50.62) | 39.50 (29.32-54.57) | 1.009(0.980-1.040) | 0.560 |
| Post-Th lymphocytes (cells/µL) | 179.55 (85.15-255.40) | 137.25 (97.22-223.08) | 0.998(0.992-1.002) | 0.339 |
| Post-Ts lymphocytes (%) | 34.85 (23.53-41.47) | 24.75 (20.65-42.05) | 0.986(0.953-1.018) | 0.406 |
| Post-Ts lymphocytes (cells/µL) | 121.30 (54.98-274.70) | 103.60 (55.07-216.70) | 1.000(0.998-1.001) | 0.824 |
| Post-CD4/CD8 | 1.04 (0.60-2.34) | 1.58 (0.79-2.49) | 1.105(0.774-1.586) | 0.580 |
| Post-NK cells (%) | 15.60 (10.33-21.68) | 19.70 (8.80-28.62) | 1.047(1.005-1.098) | 0.040 |
| Post-NK cells (cells/µL) | 70.80 (29.92-111.67) | 82.05 (35.97-147.65) | 1.002(0.998-1.007) | 0.349 |
| Post-WBC | 3.64 [2.90, 4.40] | 3.16 [2.54, 4.26] | 0.832(0.576-1.054) | 0.270 |
| Post-Lymphocytes | 0.44 [0.24, 0.62] | 0.34 [0.21, 0.51] | 0.760(0.247-2.118) | 0.608 |
| Post-Neutrophils | 2.52 [1.91, 3.25] | 2.35 [1.69, 2.91] | 0.847(0.542-1.076) | 0.372 |
| Post-Hemoglobin | 121.00 [106.00, 130.00] | 119.00 [111.50, 125.25] | 1.003(0.969-1.038) | 0.881 |
| Post-platelet | 147.00 [123.50, 183.75] | 156.50 [108.75, 193.75] | 0.998(0.990-1.005) | 0.551 |
| Post-NLR | 6.39 [3.16, 11.25] | 6.98 [3.77, 12.30] | 0.985(0.934-1.027) | 0.501 |
| Post-PLR | 68.91 [38.86, 90.16] | 76.47 [52.62, 95.15] | 1.007(0.992-1.022) | 0.361 |
| Post-SII | 24.09 [12.36, 49.66] | 20.38 [11.80, 36.63] | 1.001(0.989-1.013) | 0.833 |
| Var-Lymphocytes (10^9^/L) | -1.04 [-1.33, -0.66] | -1.15 [-1.36, -0.76] | 1.062(0.476-2.380) | 0.881 |
| Var-B lymphocytes (%) | -5.25 [-8.82, -3.78] | -5.00 [-8.10, -3.42] | 1.020(0.911-1.144) | 0.733 |
| Var -B lymphocytes (cells/µL) | -115.20 [-172.45, -71.25] | -109.70 [-132.48, -66.33] | 1.004(0.997-1.012) | 0.246 |
| Var -T lymphocytes (%) | 12.70 [3.83, 17.75] | 9.05 [2.35, 21.32] | 0.998(0.963-1.035) | 0.924 |
| Var -T lymphocytes(cells/µL) | -633.10 [-801.68, -355.20] | -638.80 [-822.70, -355.78] | 1.000(0.999-1.001) | 0.847 |
| Var -Th lymphocytes (%) | 0.05 [-9.55, 11.53] | 3.60 [-10.25, 18.12] | 1.009(0.982-1.039) | 0.505 |
| Var -Th lymphocytes (cells/µL) | -386.95 [-553.45, -292.97] | -366.40 [-518.55, -276.02] | 1.000(0.998-1.003) | 0.797 |
| Var -Ts lymphocytes (%) | 6.35 [-0.02, 17.60] | -3.30 [-2.75, 11.65] | 0.986(0.946-1.026) | 0.489 |
| Var -Ts lymphocytes (cells/µL) | -181.70 [-295.95, -89.35] | -194.55 [-342.20, -117.58] | 1.000(0.998-1.002) | 0.979 |
| Var -CD4/CD8 | -0.16 [-0.78, 0.39] | 0.13 [-0.70, 0.76] | 1.088(0.707-1.687) | 0.698 |
| Var -NK cells (%) | -6.75 [-11.13, -0.50] | -2.05 [-11.68, 5.88] | 1.024(0.983-1.070) | 0.264 |
| Var -NK cells (cells/µL) | -227.10 [-338.70, -171.85] | -291.25 [-435.05, -99.10] | 1.000(0.998-1.002) | 0.787 |
| Var-WBC | -2.71 [-3.97, -1.90] | -2.43 [-3.89, -1.25] | 0.970(0.788-1.165) | 0.742 |
| Var-Lymphocytes | -1.08 [-1.37, -0.64] | -1.19 [-1.50, -0.84] | 0.968(0.425-2.191) | 0.937 |
| Var-Neutrophils | -1.58 [-2.79, -0.84] | -1.11 [-2.34, -0.58] | 0.968(0.770-1.169) | 0.735 |
| Var-Hemoglobin | -20.50 [-28.00, -9.00] | -15.50 [-24.50, -12.00] | 1.012(0.981-1.045) | 0.464 |
| Var-platelet | -80.50 [-126.25, -48.00] | -66.50 [-127.25, -30.00] | 1.005(0.997-1.013) | 0.249 |
| Var-NLR | 2.98 [0.62, 9.30] | 4.46 [1.79, 8.83] | 0.988(0.939-1.029) | 0.572 |
| Var-PLR | 7.79 [-9.51, 26.02] | 11.32 [-12.22, 32.12] | 1.004(0.989-1.020) | 0.569 |
| Var-SII | -53.28 [-92.12, -36.56] | -61.70 [-92.88, -39.68] | 0.999(0.991-1.007) | 0.865 |
